# COMMUNITY-BASED ORGANIZATIONS CONTRACTING WITH HEALTH CARE PARTNERS TO ADDRESS SOCIAL DETERMINANTS OF HEALTH

**DOI:** 10.1093/geroni/igac059.297

**Published:** 2022-12-20

**Authors:** Robert Graham, Isha Karmacharya

**Affiliations:** Miami University, Oxford, Ohio, United States; Miami University, Oxford, Ohio, United States

## Abstract

Community Based Organizations (CBOs), such as Area Agencies on Aging, play a vital role in their communities by offering a range of services that address the health-related social needs of older adults and people with disabilities. As health care entities (HCEs) become more aware of the effects of unmet social needs on health outcomes, there has been a significant increase in the number of HCEs contracting with CBOs to provide a wider range of services and care coordination for older adults and people with disabilities. Using findings from four waves of the national Aging and Disability Business Institute’s CBO-Health Care Contracting Survey (2017–2021), presenters will describe trends in CBO/HCE contracting, including increased diversification of partnerships with health care payers and providers, services provided, and populations served. Results suggest that more CBOs are forming integrated networks to improve contracting with HCEs, and that assessment for SDOH is the most common service.

